# Neuromodulation in Spinal Cord Injury Using Transcutaneous Spinal Stimulation—Mapping for a Blood Pressure Response: A Case Series

**DOI:** 10.1089/neur.2024.0066

**Published:** 2024-09-20

**Authors:** Einat Engel-Haber, Akhil Bheemreddy, Mehmed Bugrahan Bayram, Manikandan Ravi, Fan Zhang, Haiyan Su, Steven Kirshblum, Gail F. Forrest

**Affiliations:** ^1^ Kessler Foundation, West Orange, New Jersey, USA.; ^2^ Department of Physical Medicine and Rehabilitation, Rutgers New Jersey Medical School, Newark, New Jersey, USA.; ^3^ School of Computing, Montclair State University, Montclair, New Jersey, USA.; ^4^ Kessler Institute for Rehabilitation, West Orange, New Jersey, USA.

**Keywords:** blood pressure, cardiovascular, mapping, spinal cord injury, spinal cord stimulation, spinal cord transcutaneous stimulation

## Abstract

Spinal cord transcutaneous stimulation (scTS) offers a promising approach to enhance cardiovascular regulation in individuals with a high-level spinal cord injury (SCI), addressing the challenges of unstable blood pressure (BP) and the accompanying hypo- and hypertensive events. While scTS offers flexibility in stimulation locations, it also leads to significant variability and lack of validation in stimulation sites utilized by studies. Our study presents findings from a case series involving eight individuals with chronic cervical SCI, examining the hemodynamic effects of scTS applied in different vertebral locations, spanning from high cervical to sacral regions. Stimulation of the lumbosacral vertebrae region (L1/2, S1/2, and also including T11/12) significantly elevated BP, unlike cervical or upper thoracic stimulation. The observed trend, which remained consistent across different participants, highlights the promising role of lumbosacral stimulation in neuromodulating BP.

## Background

The restoration of autonomic functions is a top recovery priority for individuals with a spinal cord injury (SCI).^1^ This includes regaining control over bowel and bladder functions, sexual capabilities, and cardiovascular (CV) regulation. Tackling CV dysfunction is crucial given its frequent presence among individuals with a high-level SCI (T6 and above). It not only hinders daily activities and diminishes quality of life but also is linked to immediate and long-term health challenges, notably increasing the risk of stroke and cardiac diseases.^2–6^ Post-SCI CV impairment is typically marked by low resting blood pressure (BP) and difficulties in stabilizing and achieving appropriate BP levels. Common complications include orthostatic hypotension (OH), a decrease in BP upon transitioning from supine to an upright position, and autonomic dysreflexia (AD), an uncontrolled elevation of BP triggered by a noxious or non-noxious stimulus.^7^

Despite various interventions, both medicinal and nonmedicinal, CV dysfunction remains a challenge without a definitive solution.^8,9^ Epidural and transcutaneous spinal cord stimulation have emerged as leading interventions in enhancing autonomic function post SCI, with recent studies providing substantial evidence supporting their safety, feasibility, and effectiveness.^10–12^ In the past, spinal stimulation research in individuals with SCI primarily focused on motor deficits,^13–16^ overlooking the significant yet often ‘invisible’ autonomic disabilities.^17^ However, in contemporary research, there is a growing recognition of the impact of these interventions in improving autonomic function, particularly in the context of CV regulation.^11,18–20^

To date, most studies on the CV effects of stimulation have utilized spinal cord epidural stimulation (scES) techniques.^20–27^ However, the inherent risks, costs, and complexity associated with spinal surgery limit the number of research participants in trials and hinder translation potential to clinical rehabilitation centers. Spinal cord transcutaneous stimulation (scTS) has emerged as an alternate noninvasive approach, with the potential to benefit large numbers of individuals and fewer risks.

A recent review examined the impact of stimulation (epidural or transcutaneous) on BP under different conditions as follows: at rest and during provocations that lead to reduced BP (causing OH) or increased BP (as seen in AD).^11^ The authors outlined the specific spinal segments targeted in stimulation, revealing a notable variability in outcomes. Stimulated spinal segments spanned from the high cervical to low sacral, demonstrating divergent, at times contradictory, results in the BP response. The majority of CV studies involving individuals with SCI utilized epidural stimulation, with the implantation of the stimulator typically occurring between vertebrae T10 to L1, targeting the lumbosacral spinal segments.^18,20,24,27,28^ While there are fewer studies of scTS-CV (transcutaneous stimulation focusing on cardiovascular function), inconsistencies become even more apparent, as there is inherent flexibility to scTS, with stimulation sites ranging from T1 to L1 vertebrae.^19,29–32^

A recurrent theme in spinal stimulation studies is the need for personalization.^33^ Key stimulus parameters such as electrode placement, frequency, and waveform pattern often demand customization due to the unique anatomical variations in the spared neural circuitry of each individual. The customization of stimulation parameters on an individual basis, known as “mapping,” is a practice usually initiated at the outset of stimulation studies. While extensively used in epidural studies, from motor to CV control,^33^ it has rarely been integrated into existing scTS studies thus far.^34^ This is understandable, given the limited number of scTS-CV studies to date;^19,31,32^ however, with the anticipated increase in the volume of these studies, the development of mapping protocols becomes essential.^17,30,35^ A recent protocol has been proposed for scTS-CV mapping, highlighting potential electrode placement sites, frequencies, amplitudes, waveforms (mono or biphasic), pulse width, and carrier frequency.^35^

The primary aim of our study is to contribute case-series data that can aid in the selection of electrode placement sites for stimulation during a mapping procedure for scTS-CV. We compare the effect of scTS applied in different vertebral locations, specifically focusing on systolic BP (SBP). Diastolic BP (DBP) and heart rate (HR) measures are also included in the article to a lesser extent. These findings can aid future research concentrating on CV functionality through transcutaneous stimulation.

## Methods

Hemodynamic data were obtained through a study that investigated the impact of scTS on upper extremity (UE) motor function (Clinical Trials Registration: NCT04921592).^34,36^ The study received approval from the Institutional Review Board at Kessler Foundation. Eligible participants were individuals aged 18 years or older, with a traumatic SCI of at least 6 months duration and a cervical injury at or above the neurological level of injury (NLI) of C8. Exclusion criteria encompassed a history of seizure disorder or malignancy, pregnancy, and current illnesses such as infection or pressure injuries that could potentially interfere with the intervention.

In the initial phase of the study, after consent was obtained and before any intervention, participants underwent a comprehensive physical and neurological examination conducted by a trained specialist in SCI. The examination included assessment of injury level (NLI) and severity (American Spinal Injury Association [ASIA] Impairment Scale [AIS]) using the International Standards for Neurological Classification of Spinal Cord Injury examination.^37^

In an early stage of the study, prior to UE testing or training, each participant underwent mapping procedures as outlined below. While UE function was the primary end-point of the mapping, CV outcomes were the secondary end-points, and hemodynamic data obtained from these sessions, i.e., continuous beat-to-beat real-time BP and HR measures, form the foundation of our study.

### scTS mapping

Each participant underwent a single day of mapping, which comprised multiple individual consecutive trials, each assessing a different electrode placement site. Stimulation was delivered utilizing a five-channel constant current stimulator (NeoStim-5, Cosyma, Moscow, Russia) at predetermined vertebral locations, including C3/4, C4/5, C5/6, C6/7, C7/T1, T1/2, T11/12, L1/2, and S1/2. Stimulation was applied one site at a time, with participants in a seated position. Self-adhesive round electrodes (2.5 cm in diameter) were placed over the midline of spinous processes at these locations as cathodes, and a pair of rectangular anode electrodes (8 × 13 cm, width x length) was positioned over the anterior iliac crests of the pelvis, following the same configuration previously reported by our team.^36^ Based on an independent methodological review,^38^ the calculated electrical characteristics for our cathode and anodes setup, respectively, are as follows: pulse charges are 40 and 25 μC, current root mean square (RMS) are 9.9 and 6.12 mA (at 30 Hz), and the current densities are 0.6 and 0.4 mA/cm^2^. The stimulation waveform consisted of a monophasic rectangular pulse with a duration of 1 ms, at a frequency of 30 Hz, and a carrier frequency of 5 kHz. The stimulation intensity was incrementally raised by 5 mA, with a time interval of approximately 5 sec between each increment. The intensity was escalated up to a maximum of 120 mA (if tolerated) in order to generate motor-evoked potentials in the majority of the UE muscles, while still staying below the motor threshold. The evoked potentials were recorded using surface electromyography (EMG); however, this procedure is detailed elsewhere as motor-evoked potentials of the UE are not pertinent to the CV mapping procedure.^39^

### BP measurements

Continuous beat-to-beat SBP, DBP, and HR measures were acquired using Finapres NOVA noninvasive blood pressure system through a single finger cuff (Finapres Medical Systems, Amsterdam, the Netherlands). Data were sampled at a rate of 1 kHz using the ADInstruments Powerlab 16/35 system (ADInstruments, Bella Vista, Australia), utilizing a Finapres Height Correction Unit to compensate for vertical movement of the hand relative to the heart level. SBP and DBP were calculated as the peaks and nadirs of the finger BP waveform, respectively. HR was calculated from the interval between two beats. Concurrently, brachial BP (Welch Allyn, Monitor 6000 Series, Skaneateles Falls, NY, USA) measures were collected at regular intervals, contralaterally to the finger cuff side. A computer interface through LabChart and custom python-based software were used to acquire all parameters synchronously and store the data.

While finger plethysmography is a well-established and reliable method to measure BP,^40^ previous studies have indicated that values may differ significantly compared with brachial measurements, particularly in cases of peripheral vasoconstriction^41,42^ or exposure to cold ambient temperatures.^43^ Consequently, an offline calibration of finger to brachial BP was performed using the following approach: For each brachial BP measure, a window was established halfway between the previous and successive measure, and a mean was calculated for the beat-to-beat finger BP measures within this window. The difference between this mean and the corresponding brachial measure was used as the offset to add to all the finger BP points within that window and calibrate them to the brachial BP. Calibration was conducted for five out of eight participants (refer to further details in the limitations section).

The primary outcome was the impact of stimulation on SBP. Utilizing the calibrated data, the collected information for participants for each stimulated location included the following:

1. Baseline SBP: Calculated as the mean of pressure points in the 10 sec preceding stimulation.
2. Maximal SBP: Analyzed using the mean of 3-sec windows during stimulation activation, with the highest mean being selected.
3. ΔSBP: Computed as the difference between maximal and baseline SBP.

In a secondary analysis, the following parameters were also examined: Baseline DBP, DBP value at the time-point of maximal SBP, and ΔDBP (the difference between the two), baseline HR, HR at the time-point of maximal SBP, and ΔHR (the difference between the two). The analysis also included the maximal amplitude, indicating the highest intensity achieved during stimulation.

### Statistical analysis

The mean, standard deviation (SD), and median of the hemodynamic measures were computed for each stimulated location. Repeated measures analysis of variance (ANOVA) was used to assess differences in means across stimulation sites for each outcome variable. In cases where significant results were obtained from the ANOVA, Bonferroni adjustments were applied for pairwise comparisons (*post hoc* analysis). Statistical significance was set at *p* < 0.05, with all *p* values considered two sided.

## Results

**FIG. 1.**
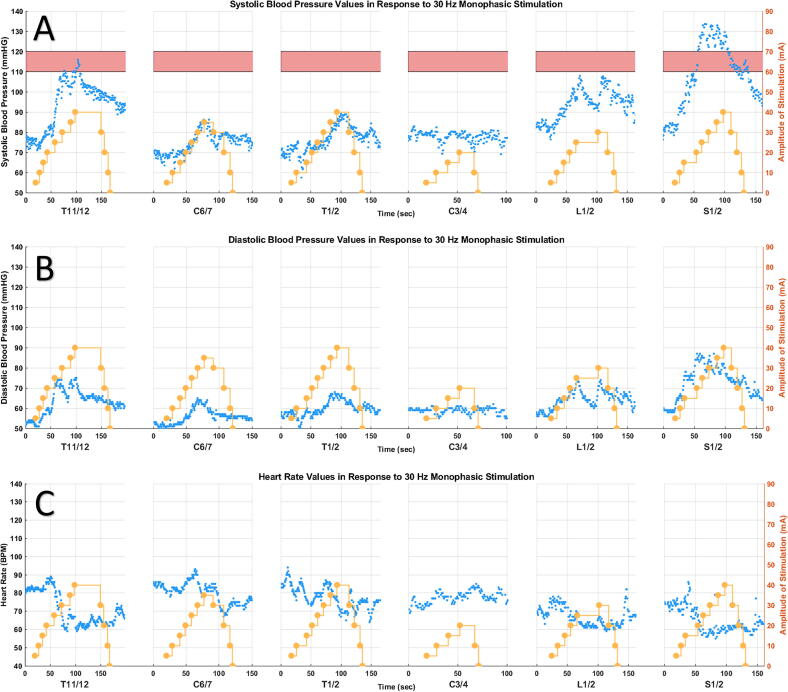
Case example for participant #1, a 24-year-old male, who sustained an AIS A C4 SCI six years ago. Monophasic stimulation was applied at a frequency of 30 Hz. The yellow dots depict stimulation intensity, pink bar represents the normotensive systolic BP range of 110–120 mmHg. The blue dots depict: **(A)** systolic BP, **(B)** diastolic BP, and **(C)** heart rate measures. Stimulation of T11/12, L1/2, and S1/2 yielded a significant increase in systolic and diastolic BP, along with a decrease in heart rate, compared with stimulation of cervical sites and T1/2. AIS, American Spinal Injury Association Impairment Scale; SCI, spinal cord injury; BP, blood pressure.

The research comprised a cohort of eight participants, all males, with chronic cervical injuries (≥6 months) (see Table 1 for participant details). Figure 1 showcases a sample of the hemodynamic data obtained for participant #1, encompassing SBP, DBP, and HR measures.

**Table 1.** Participant Characteristics

Participant	Sex	Age (years)	Time since Injury (years)	NLI	AIS
1	M	24	6	C4	A
2	M	18	7 months	C5	B
3	M	23	3	C7	B
4	M	24	3	C4	B
5	M	24	2	C5	A
6	M	35	2	C5	A
7	M	27	3	C3	A
8	M	67	1	C3	C

NLI, neurological level of injury; AIS, American Spinal Injury Association Impairment Scale.

**FIG. 2.**
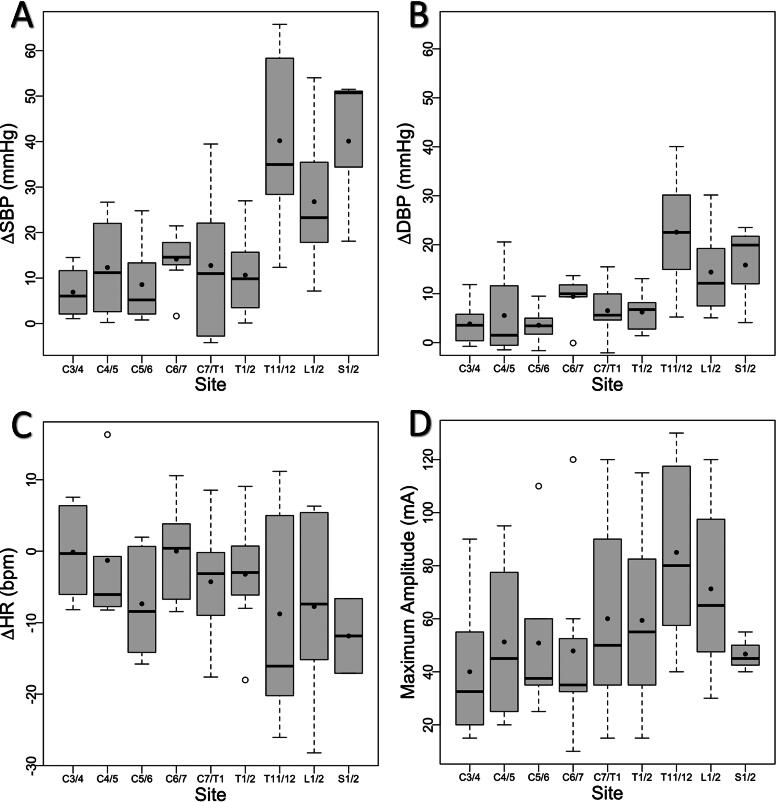
In these box plots, the boxes represent the range from the 25th to the 75th percentile, the median is shown as a horizontal line, and the average as a black dot. Any isolated dots outside the box indicate outliers, whereas the whiskers extend to cover the range within 1.5 times the interquartile range (IQR). **(A)** ΔSBP—the difference between maximal SBP with stimulation and baseline SBP prior to stimulation. **(B)** ΔDBP—the difference between DBP at the time-point of the maximal SBP with stimulation and baseline DBP. **(C)** ΔHR—the difference between HR at the time-point of the maximal SBP with stimulation and baseline HR. **(D)** Maximal amplitude of stimulation. Stimulation administered at T11/12, L1/2, and S1/2 sites elicited noticeable elevations in SBP and DBP, accompanied by a tendency for HR to decrease, compared with cervical and upper thoracic sites. SBP, systolic blood pressure; DBP, diastolic blood pressure; HR, heart rate.

Throughout the study, stimulation occurred at nine different vertebral sites (one site at a time): C3/4, C4/5, C5/6, C6/7, C7/T1, T1/2, T11/12, L1/2, and S1/2 (the latter three are considered ‘lumbosacral sites’ in this article). However, statistical analysis was conducted only for sites C3/4, T1/2, T11/12, L1/2, and C6/7, as all eight study participants received stimulation at the first four sites and seven participants (excluding participant #3) at the latter site. These sites are collectively referred to as the “five sites” in the article. Stimulation at other sites was less frequent due to the involvement of the first study participants in a pilot phase to facilitate the establishment of the protocol for UE stimulation. Stimulation at S1/2 in the first three study participants did not prove ideal for UE function and led to a significant BP increase (which was not the primary study aim) and was therefore discontinued. Later in the study, C4/5 was included as a stimulation site for four participants (#5–8). Nevertheless, we included details about all sites in the article, especially due to significant observations at S1/2, as illustrated in Figure 2.

Table 2 describes the baseline (prior to stimulation) and Δ values (change with stimulation) for SBP, DBP, and HR, as well as maximum amplitude achieved with stimulation, for the five sites.

**Table 2.** Mean (±SD) Baseline and Change with Stimulation (Δ) in SBP, DBP, and HR, as Well as Maximum Amplitude of Stimulation, for the Five Sites Included in the Analysis

	C3/4	C6/7	T1/2	T11/12	L1/2	*p* value
SBP (mmHg)	93 ± 12	93 ± 12	91 ± 14	88 ± 13*	92 ± 11*	**0.026**
ΔSBP	7 ± 5*	14 ± 6	11 ± 9	40 ± 19*	27 ± 15	**<0.0001**
DBP (mmHg)	69 ± 9	65 ± 7	66 ± 10	61 ± 9	67 ± 7	**0.0066**
ΔDBP	4 ± 4*	9 ± 5	6 ± 4	23 ± 11*	14 ± 10	**0.0002**
HR (bpm)	73 ± 10	73 ± 7	83 ± 13	75 ± 10	75 ± 8	0.107
ΔHR	0 ± 7	0 ± 7	−3 ± 8	−16 ± 15	−7 ± 13	0.869
Maximum Amplitude (mA)	40 ± 26	48 ± 35	60 ± 34	85 ± 34	71 ± 31	**0.0023**

Bold highlights in the *p* value column indicate significant findings in the analysis of variance (ANOVA) among the five sites. Significance (*p* < 0.05) in *post hoc* pairwise analysis is denoted by an asterisk (*).

SD, standard deviation; SBP, systolic blood pressure; DBP, diastolic blood pressure; HR, heart rate.

### SBP

The average baseline SBP among the five sites was largely consistent, ranging between 88 and 93 mmHg, although a repeated measures ANOVA revealed some variance (*p* = 0.026). Subsequent *post hoc* analysis identified a significant difference only between the T11/12 and L1/2 sites (*p* value = 0.033). Figure 2A presents the increase in SBP with stimulation (ΔSBP). Upon observation, the lumbosacral sites (L1/2, S1/2, T11/12) exhibited a notable rise in SBP with stimulation. In a repeated measure ANOVA analysis combining the five sites, differences were observed (*p* < 0.0001); however, *post hoc* tests pointed out a significant difference only between C3/4 and T11/12 (*p* = 0.038), likely due to the small sample size.

### DBP

Similar trends to SBP were observed. While there were some differences in baseline DBP across the five sites, ranging from 61 to 69 mmHg (*p* = 0.0066), Bonferroni multiple comparisons did not reveal significant differences. When evaluating the change in DBP, on visual inspection, the lumbosacral sites showed a significant DBP increase with stimulation compared with cervical sites (Fig. 2B). The comparison of ΔDBP among the five sites was significant (*p* = 0.0002). Akin to ΔSBP, *post hoc* analysis only showed significant differences between C3/4 and T11/12 (*p* = 0.026).

### HR

Data on HR were limited, as it was only partially recorded compared with SBP and DBP. With only four observations for the five sites, a repeated measures ANOVA analysis did not detect significant differences in baseline or ΔHR (Fig. 2C) among the five sites. However, despite the lack of significance, in most stimulated sites, the average ΔHR was <0, indicating that at the point of maximal SBP increase (with stimulation), HR simultaneously decreased.

### Maximum amplitude

In terms of the maximum amplitude (intensity) applied across the various stimulation sites, on visual inspection, lower stimulation intensities were used in the cervical sites compared with lumbosacral (Fig. 2D). Our analysis indicated a significant difference among the five sites (*p* = 0.0023); however, *post hoc* analyses did not find a significant difference between any pair.

## Discussion

CV dysfunction occurs at two ends of the BP spectrum as follows: AD, characterized by uncontrolled high BP, and OH, marked by unstable low BP. Previous research has shown the efficacy of both epidural and transcutaneous (scTS) stimulation in alleviating these conditions. In this investigation we examined the hemodynamic effects induced by scTS across diverse electrode placement sites, in individuals with SCI who have low baseline BP, to identify potential locations that could elicit a notable BP response—specifically, a controlled increase in SBP in response to stimulation.

Despite the anticipated variability across participants, our findings reveal a persistent trend as follows: stimulation of lumbosacral sites (specifically, between vertebrae T11/12, L1/2, or S1/2) consistently resulted in an elevation of BP compared with cervical and higher thoracic sites. These results can inform future scTS-CV studies addressing autonomic impairment in individuals with SCI.

There is a significant variation in the choice of electrode placement sites in the few scTS-CV studies conducted to date. Phillips et al. carried out research with five subjects who have SCI and confirmed OH. ScTS was applied at the T7/8 vertebral level to regulate BP and alleviate orthostatic symptoms during an orthostatic provocation.^19^ Sachdeva et al. utilized the same location to showcase the effectiveness of scTS in preventing and treating AD induced by a digital anorectal stimulation procedure in one individual.^29^ In a separate case series involving three patients with orthostatic issues caused by autonomic dysfunction (not SCI), scTS was simultaneously applied at T1, T5, and T7 to restore BP.^31^ In a recent investigation by Solinsky et al., the effects of scTS on autonomic control were explored, with stimulation targeting the T10/11 site.^32^

Spatiotemporal mapping techniques, utilized to guide electrode placement and stimulation parameters, have not been widely utilized in transcutaneous stimulation (CV and non-CV), although being extensively used in epidural stimulation.^33^ This is evident from the scTS studies mentioned earlier, using the same vertebral level for stimulation for every participant within each respective study.^19,29,31,32^ Nevertheless, the increasing number of scTS studies highlights the necessity for a standardized approach to determine the best configurations and facilitate study comparisons.^35^

Ongoing efforts are directed toward developing study protocols for scTS mapping with a specific focus on motor control and autonomic function.^17,30,35^ Our latest work utilized mapping techniques to enhance upper extremity function through scTS.^34^ Moreover, a recent protocol for scTS-CV mapping recommended testing the following sites for electrode placement: T7/8, T9/10, T11/12, and L1/2, with the goal of increasing and stabilizing BP within the target range.^35^ Our study included the T11/12 and L1/2 sites, both of which demonstrated a significant BP response. However, certain thoracic sites, including T7/8, were not included in this investigation.

### Mechanisms

The general paradigm suggests that stimulation (whether through scES or scTS) delivers electrical currents to the afferent sensory fibers through the posterior roots to engage the sympathetic circuitry.^12,20^ This mechanism involves a somato–autonomic reflex (a reflex induced by sensory afferent inputs and manifesting as an alteration of autonomic nervous system function), which may excite or inhibit sympathetic pre- and postganglionic neurons.^12^ Various hypotheses exist regarding the optimal stimulation location and the underlying mechanisms guiding this choice.

Our finding of a significant BP response when electrodes are placed at T11/12 or L1/2 aligns with previous epidural studies, where the electrodes were typically implanted at T10-L1 vertebrae levels, corresponding to the lumbosacral spinal levels.^18,21,22,24,25,27,28^ It is important to highlight that scTS research consistently specifies the vertebral sites for electrode placement, in contrast to scES studies, which often identify the spinal segment targeted. This difference arises from the following distinct techniques used: scES utilizes an implantable 16-electrode array, facilitating a more detailed depiction of the stimulation area. For example, in a recent scES study, Angeli et al. suggested focusing on spinal cord levels L3 and L4 for CV regulation.^33^ ScTS, on the other hand, is not as specific as scES and depends on electrode sizes, which influence the size of the electrical fields.

Some studies advocate for stimulation at higher locations, proposing that direct targeting of sympathetic preganglionic neurons (SPNs), situated between T1 and L2, through afferent inputs, is optimal for a BP response. In their scTS studies, Phillips and Sachdeva targeted T7/8, “where sympathetic preganglionic neuron cell bodies are located”.^19,29^ In an scES study by Squair et al., the T10–T11 posterior root area was identified as an anatomical and functional hotspot, containing the highest density of SPNs. This region was chosen for stimulation in one individual.^20^

However, this body of evidence falls short in explaining the notable BP increase observed in our study and epidural studies that specifically target lower spinal segments, where SPNs are no longer present. In this scenario, it is postulated that stimulation activates propriospinal neurons through interneuron-based relays, ultimately influencing the SPNs and resulting in blood vessel constriction and normalization of BP.^28,44^ Although this study did not apply stimulation to model the electrical field distributions through the spinal regions, it has been reported that the electrode positioning changes the outcome, especially for the lower regions, for both scTS^45,46^ and scES.^47,48^ Fernandes et al.^46^ suggested that the anatomy of the spine, mainly the highly conductive cerebrospinal fluid volume, is directly and negatively correlated with the electrical field, which may lead to a larger current in the lower regions that could go paravertebrally. Thus, even with minimal spinal cord presence, the electrical field distribution has the potential to spread. Therefore, the paravertebral sympathetic chain ganglia, comprising of the thoracic, lumbar, and sacral ganglia, is potentially within the field of stimulation for the lumbosacral cathode sites and the bilateral anterior superior iliac spine (ASIS) anodes for autonomic modulation effect.^49–51^

The limited response to cervical site stimulation that was observed in our study may be partly ascribed to the use of lower intensities during stimulation (see Fig. 2D), a measure taken in response to participant discomfort, which was often more pronounced with cervical stimulation.^52^ Nevertheless, even in certain cases where higher intensities were administered to participants capable of tolerating them, a significant BP response in cervical sites was generally not observed. In SCI research, cervical stimulation is typically used to improve upper extremity function. Interestingly, it has been shown to also have remote effects, leading to improvements in lower extremity movement.^52–54^ However, BP was not an outcome measure in these cervical stimulation studies and was only monitored for safety. While some studies reported a sporadic increase in BP with cervical stimulation, it was not consistent or significant.^55–57^

The substantial increase in BP observed with stimulation at the vertebra level of S1/2 raises questions about the underlying mechanism, as there is no longer cord at that level. Despite limited data in our study from only three participants stimulated at S1/2, the BP response at this site was significant, even with a relatively low stimulation intensity. This is somewhat similar to several recent scTS studies focusing on locomotion that demonstrated that the addition of a coccygeal site for stimulation enhances the stimulation effect, although the underlying mechanism remains unclear and is likely different.^15,47,52^

A potential explanation is that stimulation at S1/2 may result in retrograde neuromodulation, suggesting a relay to the spinal cord through peripheral spinal nerves—a mechanism akin to the approach seen in posterior tibial nerve stimulation.^58^ One must not rule out the possibility that stimulation at this specific location elicited an AD response or a substantial increase in venous return due to leg muscle contraction. Either of these mechanisms could contribute to the observed increase in BP.

The hypothesis that an AD response may contribute to the observed BP increase in stimulation of other lumbosacral sites, not solely limited to S1/2, appears plausible. This is further supported by the decrease in HR observed in most participants with stimulation of these sites, as depicted in Figures 1 and 2, aligning with the typical “classical” pattern seen in AD (although tachycardia is also often seen).^59,60^ Nevertheless, this HR response was not consistent across participants, with instances where participants showed an increase in HR alongside a BP increase (not depicted). This was seen in other stimulation studies, where alongside a BP increase, HR exhibited varied behavior; it either remained stable, decreased, or increased with stimulation.^18,19,24^ Therefore, one should not solely rely on the HR response to differentiate between AD and other phenomena.

Despite the observed elevation in BP among study participants, there were no reported adverse symptoms indicative of an AD response. Further factors that demonstrate this is not a typical AD reaction include a theory suggesting that stimulation activates large-diameter sensory axons which do not typically trigger AD.^19^ Moreover, the observed increase in BP with stimulation in our study was controlled and moderate, deviating from the typical pattern associated with AD.^19,25^

Studies have shown that stimulation (both epidural and transcutaneous) not only effectively reduces the severity of AD episodes but also can prevent them.^28,29^ This suggests that stimulation has the ability to regulate autonomic function, providing relief from OH through the activation of sympathetic efferent pathways and mitigating AD through inhibitory mechanisms.^12^ Nevertheless, Solinsky et al. recently proposed that scTS may not improve autonomic regulation after SCI. Instead, they postulate that it generates tonic, frequency-dependent sympathoexcitation, which may lower the threshold for AD.^32^ Further study is needed to elucidate the mechanisms behind the BP response to stimulation and its relation to direct sympathetic activation versus indirect activation through sensory perception of a noxious stimulus or extraspinal pathways.

The increase in BP observed with lumbosacral stimulation could also potentially be attributed to the contraction of lower leg muscles, resulting in an elevated venous return and subsequent BP rise. Previous studies used EMG to investigate the involvement of lower extremity muscles and explored configurations that allow for an increase in BP with stimulation without engaging skeletal muscle pump action.^18,19^ These studies provide evidence that skeletal muscle pump action is not the cause of the pressor response. Conversely, Aslan et al. observed an increase in EMG activity in leg muscles, but the modulation of BP was evident only in individuals with CV dysfunction.^24^ Since our primary study focused on upper extremities, we do not have EMG data of the lower limbs.

### Considerations for future studies on BP modulation

This study specifically examined the impact of stimulating various vertebral locations. However, there are additional parameters involved in a mapping procedure not emphasized in this discussion.^35^ The frequency of 30 Hz, employed in our study, is commonly used in CV stimulation studies using both epidural and transcutaneous approaches.^18,19,31^ Nonetheless, other frequencies, such as 2 Hz,^24^ 50 Hz,^18^ 60 Hz,^35^ and 120 Hz,^20,32^ have also been explored. The determination of which frequencies yield optimal results in terms of BP modulation remains an open question that warrants further investigation. Higher frequencies have been suggested for CV-related stimulation studies due to the observed reduced motor activation.^33^ Besides frequency modulation, other parameters like pulse width, waveform (mono or biphasic), and carrier frequency, not described in this study, could be modulated. In this study, we used a monophasic waveform, consistent with our prior scTS UE research^36^ and other studies.^61^ Contrary to initial expectations,^62^ our laboratory found that monophasic stimulation was better tolerated by participants for the upper extremity research. Further study is needed to compare the efficacy and safety of mono- and biphasic stimulation, as biphasic stimulation, which utilizes charge balancing, is likely safer for prolonged use.^62^

As this investigation constituted a secondary analysis of BP measures, our primary aim did not entail achieving SBP stability within the normotensive target range of 110–120 mmHg,^18^ as pursued in other CV stimulation studies.^18,33,35,63^ Consequently, our study does not center on the proportion of measures within this range; rather, our emphasis is on assessing our ability to induce an increase in BP. For future mapping studies with a primary emphasis on both increasing and stabilizing BP within a specified range, it becomes imperative to incorporate additional metrics assessing BP stability. One recent clinimetric approach involved the use of an exponential distribution function to evaluate BP stability within the target range.^64^ There is a clear need for additional research to further explore and refine these measures.

### Limitations

There are certain implications stemming from the fact that CV was not the primary outcome in this study. First, there are no EMG data of the lower limbs, and therefore, it is possible that some of the BP increase was due to contractility of the lower extremities and increased venous return. Of note, during stimulation, the investigators did not observe muscle contractions in the lower limbs. Our laboratory is now conducting additional research to evaluate lower limb activity when stimulating for a CV response. Moreover, only certain sites were investigated in relation to upper extremity function. Therefore, more cervical sites were assessed, but T7/8, a site used by previous transcutaneous studies,^19,29^ was not included.

The offline calibration of finger with brachial BP, as detailed in the Methods section, was carried out for only five out of the eight individuals. This limitation arose because time stamps of brachial BP were not collected for the initial three participants. In future studies, calibration will be conducted for all participants. However, given our primary focus on the change (ΔSBP) and less on baseline values, this calibration has minimal impact.

In the mapping procedure, there’s inherent variability, with each mapping session comprising several consecutive trials that test different sites. Notably, baseline BP in the initial trials of a session was sometimes lower compared with later trials within the same session. This discrepancy could be attributed to a carryover effect or adaptation of the BP response, potentially priming it to be more responsive to stimulation.

## Conclusions

This case series illustrated that transcutaneous stimulation at lumbosacral vertebral levels consistently elevated BP in individuals with chronic SCI, in contrast to stimulation of cervical and higher thoracic sites. Additional research is necessary to unveil the mechanism behind this phenomenon and explore alternative configurations that could contribute to ameliorating cardiovascular dysfunction in individuals with SCI.

## Abbreviations Used

AD: autonomic dysreflexia
AIS: ASIA impairment scale
ASIA: American spinal injury association
ASIS: anterior superior iliac spine
BP: blood pressure
CV: cardiovascular
DBP: diastolic blood pressure
EMG: electromyography
HR: heart rate
NLI: neurological level of injury
OH: orthostatic hypotension
RMS: root mean square
SBP: systolic blood pressure
SCI: spinal cord injury
scES: spinal cord epidural stimulation
scTS: spinal cord transcutaneous stimulation
scTS-CV: scTS targeting cardiovascular
SPN: sympathetic preganglionic neuron
UE: upper extremity
